# Oral eltanexor treatment of patients with higher-risk myelodysplastic syndrome refractory to hypomethylating agents

**DOI:** 10.1186/s13045-022-01319-y

**Published:** 2022-08-03

**Authors:** Sangmin Lee, Sanjay Mohan, Jessica Knupp, Kamal Chamoun, Adrienne de Jonge, Fan Yang, Erkan Baloglu, Jatin Shah, Michael G. Kauffman, Sharon Shacham, Bhavana Bhatnagar

**Affiliations:** 1 Present Address: Division of Hematology and Oncology, Weill Cornell Medicine, The New York Presbyterian Hospital, 520 East 70thStreet, Starr 341, New York, NY 10021 USA; 2 Division of Hematology and Oncology, Vanderbilt University Medical Center, Nashville, TN USA; 3 Karyopharm Therapeutics Inc, Newton, MA USA; 4 West Virginia University Cancer Institute, Wheeling Hospital, Wheeling, WV USA; 5 Present Address: Janssen Research and Development, Spring House, PA USA

## Abstract

Patients with higher-risk myelodysplastic syndromes (MDS) refractory to hypomethylating agents (HMAs) have limited therapeutic options and an expected overall survival (OS) of 3–5 months. Eltanexor is an investigational oral selective inhibitor of nuclear export with low central nervous system penetrance and an acceptable tolerability profile. Preclinical studies suggest that myeloid malignancies are sensitive to nuclear export inhibition. Eltanexor exhibited efficacy in hematologic models, supporting exploration in a clinical trial. This phase 1/2 study (NCT02649790) assessed single-agent activity of eltanexor in patients with higher-risk MDS and 5–19% myeloblasts. Two starting doses of eltanexor were evaluated: 20 mg (*n* = 15), 10 mg (*n* = 5), both administered on days 1–5 each week of a 28-day cycle. Twenty patients with primary HMA-refractory MDS, with a median age of 77 years (range 62–89), and a median of two prior treatment regimens (range 1–4) were enrolled. Of these, 15 were evaluated for efficacy and 20 for safety. The overall response rate (ORR) was 53.3%, with seven patients (46.7%) achieving marrow complete remission (mCR) and one additional patient achieving hematologic improvement (HI). In the 10 mg group, three patients (60%) reached mCR and two (40%) stable disease (SD), while for 20 mg, four patients (40%) had mCR and two (20%) SD. A total of three patients (20%) had HI and became transfusion independent ≥ 8 weeks. Median OS for the efficacy-evaluable patients (*n* = 15) was 9.86 months (7.98, NE). Overall, the most frequently reported treatment-related adverse events were nausea (45%), diarrhea (35%), decreased appetite (35%), fatigue and neutropenia (both 30%). Single-agent oral eltanexor was active, safe, and well tolerated in patients with higher-risk, primary HMA-refractory MDS.

**Supplementary Information:**

The online version contains supplementary material available at 10.1186/s13045-022-01319-y.

## To the Editor:

Higher-risk myelodysplastic syndrome (MDS) carries a significant risk of progression to acute myeloid leukemia (AML) and decreased survival [1]. Hypomethylating agents (HMAs) are first-line therapies for this population, demonstrating tolerability and improved overall survival (OS) [2–5]. However, HMAs are not curative for MDS, and thus, treatment of refractory or relapsed higher-risk MDS patients after HMA therapy represents a profound unmet medical need, with no approved therapeutics and median OS (mOS) of 4–5.6 months [6, 7].

Selective inhibitor of nuclear export (SINE) compounds are a class of novel and specific antagonists of the karyopherin exportin 1 protein (XPO1). Eltanexor is a reversible, oral SINE compound, which in non-clinical AML mouse studies showed robust single-agent activity, with improved tolerability and overall survival [8, 9]. We therefore analyzed eltanexor treatment in a phase 1 cohort of patients with higher-risk, HMA-refractory MDS.

Twenty patients with MDS were treated with single-agent eltanexor at a starting dose of 20 mg (*n* = 15) or 10 mg (*n* = 5) on days 1–5 of each week of each 28-day cycle (Additional file 1: Figure S1). The majority of patients were aged ≥ 75 years with de novo MDS, and all patients had MDS primary refractory to HMAs (Additional file 1: Table S1).

Of the 20 patients, seven (35%) had marrow complete remission (mCR), including two patients that also achieved hematologic improvement (HI). One (5%) additional patient had HI and stable disease (SD), and four patients (20%) had SD. Overall, the disease control rate (DCR) was 60% (95% CI 36.1–80.9), and the overall response rate (ORR) was 40% (95% CI 19.1–64.0). Of the 20 patients enrolled, 15 patients were evaluable for efficacy, as five 20 mg patients discontinued the study and were thus not evaluable for disease response; ORR for the efficacy-evaluable patients was 53.3% (95% CI 26.6–78.7; Table 1).

**Table 1:** Efficacy in evaluable patients

	Eltanexor (10 mg) *N* = 5	Eltanexor (20 mg) *N* = 10	Total *N* = 15
ORR, *n* (%)	3 (60)	5 (50)	8 (53.3)
MCR, *n* (%)	3 (60)	4 (40)	7 (46.7)
HI, *n* (%)	1 (20)	2 (20)	3 (20)
HI with MCR	1 (20)	1 (10)	2 (13.3)
HI with SD	0	1 (10)	1 (6.7)
SD, *n* (%)	2 (40)	2 (20)	4 (26.7)
PD, *n* (%)	0	3 (30)	3 (20)
Treatment duration, weeks	15	12	13
Median time to response, weeks	8.1	9.1	8.4
Median duration of response, weeks (95% CI)	7.86 (NE, NE)	25.29 (13.12, NE)	19.21

*ORR* overall response rate; *MCR* marrow complete remission; *HI* hematologic improvement; *SD* stable disease; *PD* progressive disease; *CI* confidence interval; * NE* not evaluable

Three patients (20%) had HI and became transfusion independent ≥ 8 weeks. Among patients on eltanexor at the 20 mg dose, four patients (40%) reached mCR, including one patient who reached mCR with HI, one patient (10%) had HI and SD, and two (20%) had SD. Of patients on the 10 mg dose, three patients (60%) reached mCR, including one patient who had mCR with HI, and two patients had SD (40%).

**Fig. 1:**
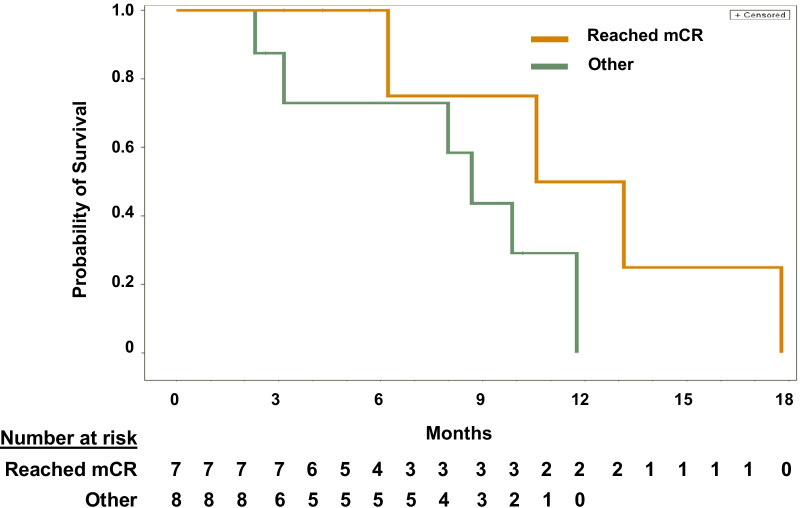
Overall Survival. The median OS for all patients was 9.86 months. OS was higher in mCR patients (*n* = 7) than patients who did not reach mCR (*n* = 8): median 11.86 vs 8.67 months (HR = 0.28, *p* = 0.11), and longer than OS for patients with PD (*n* = 3, mOS = 3.15 months, HR = 0.23, *p* = 0.08). mCR = marrow complete remission; OS = overall survival; HR = hazard ratio; PD = progressive disease

After 3–4 cycles of treatment, levels of normal neutrophils and platelets improved, while blasts decreased, and patients had a median reduction in bone marrow blasts from baseline of -78.2% (range −86% to 33%; Additional file 1: Figure S2). mOS for the entire efficacy-evaluable cohort was 9.86 months. OS for patients who reached mCR was longer than for those who did not reach mCR, with a median of 11.86 vs 8.67 months, respectively (HR = 0.28, p = 0.11; Fig. 1). Additionally, mOS for patients with mCR was longer than mOS for patients with progressive disease (PD; 3.15 months [HR = 0.23, *p* = 0.08]), and patients with SD had longer OS than patients with PD (9.86 vs 3.15 months, HR = 0.38, *p* = 0.09).

None of the patients experienced dose-limiting toxicities (DLTs). The most frequently occurring adverse events (AEs) of any grade were non-hematologic and occurred more often in the 20 mg treatment group than the 10 mg group (Additional file 1: Table S2). Serious AEs (grade 3/4) were infrequent, and there were no grade 5 events. Of the 20 patients initially treated with eltanexor, seven patients (35.0%) required a dose delay, and eight patients (40.0%) required a dose reduction to 10 mg due to adverse events.

Novel agents to treat relapsed/refractory MDS patients are currently in development, though recent phase 3 trials to develop novel treatments for higher-risk MDS patients were unsuccessful, failing to meet the primary endpoints of improving OS [10, 11]. All patients in this study had MDS primary refractory to HMAs, 19/20 were considered higher risk by IPSS scoring, and 25% of patients had poor risk cytogenetics. Despite these factors, the ORR was still 53.3% in efficacy-evaluable patients, demonstrating an encouraging initial response to eltanexor monotherapy. Consistent with the selectivity of eltanexor for leukemic cells, responses included both a substantial reduction in blasts and commensurate increases in normal neutrophils and platelets, even for patients who achieved SD rather than a response [12]. A limitation of this report is the small patient population.

Overall, eltanexor represents a mechanistically novel, oral agent with good tolerability and robust single-agent activity conferring improved survival relative to historical controls, supporting the continued development of this novel agent.

## Supplementary Information

**Additional file 1.** Methods, supplementary tables S1 and S2, and supplementary figures S1 and S2.

## Data Availability

The datasets used and/or analyzed during the current study are available from the corresponding author on reasonable request.

## Abbreviations

AE: Adverse event
AML: Acute myeloid leukemia
CI: Confidence intervals
DLT: Dose-limiting toxicity
DOR: Duration of response
HI: Hematologic improvement
HMA: Hypomethylating agent
HR: Hazard ratio
IPSS: International Prognostic Scoring System
mCR: Marrow complete response
MDS: Myelodysplastic syndromes
mOS: Median overall survival
NE: Not evaluable
ORR: Overall response rate
OS: Overall survival
PD: Progressive disease
SD: Stable disease
SINE: Selective inhibitor of nuclear export
XPO1: Exportin 1
