# Supplementary material for: Oral eltanexor treatment of patients with higher-risk myelodysplastic syndrome refractory to hypomethylating agents
Source: J Hematol Oncol. 2022 Aug 3;15:103. doi: 10.1186/s13045-022-01319-y (PMC9351096; doi:10.1186/s13045-022-01319-y)
Supplement: Supplementary file 1 — Additional file 1. Methods, supplementary tables S1 and S2, and supplementary figures S1 and S2. [file 13045_2022_1319_MOESM1_ESM.docx]

**Oral Eltanexor Treatment of Patients with Higher-Risk Myelodysplastic Syndrome Refractory to Hypomethylating Agents**

Sangmin Lee, MD;^1ǂ2*^ Sanjay Mohan, MD;^3^ Jessica Knupp, MPH;^4^ Kamal Chamoun, MD;^4^ Adrienne de Jonge, RN;^4^ Fan Yang, PhD;^4^ Erkan Baloglu, PhD;^4^ Jatin Shah, MD;^4^

Michael G. Kauffman, MD, PhD;^4^ Sharon Shacham, PhD, MBA;^4^ Bhavana Bhatnagar, DO^5^

**Methods**

***Study Design and Participants***

This phase 1/2, multicenter, open-label, dose-escalation, and expansion study is designed to evaluate oral eltanexor in patients with relapsed or refractory higher-risk MDS, multiple myeloma, metastatic colorectal cancer, metastatic castration-resistant prostate cancer, and AML (NCT02649790). This report is on the results for the phase 1 higher-risk, HMA-refractory MDS cohort which was studied at sites in the US and Canada. Patients ≥18 years with histologically confirmed higher-risk MDS and 5-19% myeloblasts (MDS-EB-2 by the 2016 World Health Organization classification) were enrolled.^24^ Higher-risk MDS was defined as intermediate-2, or high risk MDS, by International Prognostic Scoring System (IPSS) criteria.^25^ Patients with Eastern Cooperative Oncology Group (ECOG) performance status of 0-2 and de novo or secondary MDS were included. Patients were required to have HMA-refractory disease, defined as having progressive disease (based on 2006 International Working Group [IWG] Response Criteria for MDS)^26^ after receiving ≥2 cycles of azacitidine and/or decitabine or experimental agents, or no response after ≥4 cycles of any HMA. Eligible patients were required to have adequate hepatic (total bilirubin ≤2 x upper limit of normal, aspartate aminotransferase and alanine aminotransferase ≤2.5 x upper limit of normal), and renal function (creatinine clearance ≥30 mL/min).

Patients were excluded from the study if they had disease transformation to AML, concomitant or previous malignancies with a <1-year disease-free interval at enrollment, active central nervous system malignancy, or gastrointestinal disease or disruption (uncontrolled vomiting or diarrhea) that could interfere with the absorption of eltanexor. Patients who received granulocyte-colony stimulating factor or granulocyte macrophage-colony stimulating factor within three weeks of cycle one day one (C1D1) were also excluded from the MDS cohort. The dose-limiting toxicity (DLT) observation period was cycle 1. A DLT was defined as ≥grade 3 nausea/vomiting while taking optimal supportive medications, or any other ≥grade 3 non-hematological toxicity, except alopecia or electrolyte abnormalities, correctable with supportive therapy.

The initial dose (20mg, once daily, five days on, two days off per week of each 28-day cycle) used in the first 15 patients was selected based on a dose escalation phase in patients with relapsed/refractory multiple myeloma that identified 20mg as the recommended phase two dose. Though no formal DLTs were demonstrated, eight of the 15 patients (53.3%) required dose reduction to 10mg during treatment, and the Investigators and Sponsor’s Medical Monitor reviewed the available safety data and agreed to enroll a cohort of patients (n=5) at a starting dose of 10mg (once daily, five days on, two days off per week of each 28-day cycle) as a strategy to improve tolerability.

Bone marrow aspirates and biopsies were completed at screening, cycle three day one (C3D1), C7D1, C13D1, and, when possible, at the time of disease progression. In addition to safety and tolerability assessment, preliminary efficacy endpoints included overall response rate (ORR), DOR, progression-free survival (PFS), overall survival (OS), disease control rate (DCR), duration of DCR, and pharmacokinetics of eltanexor in patients with higher-risk MDS.

The study protocol was approved by the institutional review board or an independent ethics committee at each participating center and is in accordance with the Declaration of Helsinki, International Conference on Harmonization-Good Clinical Practice, and local laws. Written informed consent was obtained from all patients before enrollment.

***Outcomes***

Safety and tolerability of eltanexor were determined by adverse event (AE) reports, physical examinations, electrocardiograms, and laboratory safety evaluations. Adverse events were graded using Common Terminology Criteria for Adverse Events version 4.03. Disease response was evaluated by the treating physician according to the 2006 International Working Group Response Criteria for MDS.^26^ ORR included CR, partial response (PR), marrow complete response (mCR), and hematologic improvement (HI). DOR was defined as the time from first meeting the ORR criteria until progressive disease (PD) or recurrence. PFS was the time from first dose of study treatment until PD or death due to any cause. OS was time from first dose of study treatment until death due to any cause. DCR was the sum of ORR and rate of SD, and duration of DCR was the time from first meeting the DCR criteria until PD or recurrence.

***Statistical Analysis***

The sample size was calculated using a 2-stage design, where the null hypothesis would be ORR ≤10% against a one-sided alternative that the true ORR is ≥35%, using a type I error of 0.1 (one-sided) and a power of 80%. Twelve patients were enrolled in Stage 1. If ≥3 patients respond, then eight additional patients were enrolled into Stage 2. If a total of five of the 20 patients combined across both stages respond, the treatment was accepted as promising for further study. Descriptive statistics were used to summarize safety and efficacy endpoints by dose cohort. For categorical variables, summary tabulations of frequency and percentage of patients within each category were presented, along with two-sided 95% exact confidence intervals (CI) where appropriate. For continuous variables, the number of patients, mean, median, standard deviation, minimum, and maximum values were presented. Time-to-event data were summarized using Kaplan-Meier methodology with associated 2-sided 95% CIs.

**SUPPLEMENTAL TABLES**

**Table S1 Baseline Characteristics**

| **Characteristic** | **Total**  **N=20** |
| --- | --- |
| **Age** |  |
| Years, median (range) | 77 (62 - 89) |
| <75, n (%) | 7 (35) |
| ≥75, n (%) | 13 (65) |
| **Sex, n (%)** |  |
| Male | 11 (55) |
| Female | 9 (45) |
| **Race, n (%)** |  |
| White | 13 (65) |
| Black or African American | 1 (5) |
| Asian | 1 (5) |
| Other/Not Reported | 5 (25) |
| **Eastern Cooperative Oncology Group Performance Status, n (%)** |  |
| 0 | 4 (20) |
| 1 | 13 (65) |
| 2 | 3 (15) |
| **International Prognostic Scoring System**  **Risk Score, n (%)** |  |
| Intermediate-1 | 1 (5) |
| Intermediate-2 | 7 (35) |
| High | 12 (60) |
| **MD Anderson Cancer Center Risk Score, n (%)** |  |
| Intermediate-1 | 2 (10) |
| Intermediate-2 | 13 (65) |
| High | 5 (25) |
| **Time Since Initial Diagnosis of MDS** |  |
| Years, median (range) | 2.5 (0.6 – 8.9) |
| **MDS Subtype, n (%)** |  |
| De Novo | 16 (80) |
| Secondary | 4 (20) |
| **Prior Therapies, n (%)** |  |
| Median (range) | 2 (1 - 4) |
| Azacitidine | 14 (70) |
| Decitabine/ASTX727 | 11 (55) |
| Lenalidomide | 4 (20) |
| Chemotherapy | 2 (10) |
| Investigational Agent | 3 (15) |
| Other | 4 (20) |
| **Blood Counts on Cycle 1 Day 1, median (range)** |  |
| Platelets (K/µL) | 26 (12–88) |
| Hemoglobin (g/dL) | 8.3 (7.0–9.3) |
| Neutrophils (K/µL) | 1.2 (0.1–9.0) |
| **Bone Marrow Blasts %** **on Cycle 1 Day 1, median (range)** | 10 (7-18) |
| **Mutational Status*, n (%)** |  |
| TET2 | 8 (40) |
| ASXL1 | 7 (35) |
| EZH2 | 4 (20) |
| DNMT3A | 3 (15) |
| TP53 | 2 (10) |
| KRAS | 2 (10) |
| IDH1 | 2 (10) |
| N-RAS | 1 (5) |
| SF3B1 | 1 (5) |
| Normal | 5 (25) |
| **Cytogenetic Risk Group*, n (%)** |  |
| *Good* |  |
| Normal | 8 (40) |
| Del (5q) | 1 (5) |
| *Intermediate* |  |
| Del(16q) | 1 (5) |
| 21+ | 1 (5) |
| *Poor* |  |
| Del(7q) | 3 (15) |
| Complex Karyotype | 5 (25) |
| *Unknown* |  |
| Insufficient | 1 (5) |

*Patients may have more than one mutation or cytogenetic abnormality.

MDS = myelodysplastic syndromes

**Table S2 Treatment-Related Adverse Events in ≥10% of Patients**

| **Adverse Event, n (%)** | **10mg Eltanexor (N = 5)** | | | | **20mg Eltanexor (N = 15)** | | | |
| --- | --- | --- | --- | --- | --- | --- | --- | --- |
| **Non-Hematologic** | **G1** | **G2** | **G3** | **G4** | **G1** | **G2** | **G3** | **G4** |
| Nausea | - | - | - | - | 4 (26·7) | 5 (33·3) | - | - |
| Decreased Appetite | - | - | 1 (20·0) | - | 3 (20·0) | 3 (20·0) | - | - |
| Diarrhea | 2 (40·0) | - | - | - | 3 (20·0) | 1 (6·7) | 1 (6·7) | - |
| Fatigue | - | 2 (40·0) | - | - | - | 2 (13·3) | 2 (13·3) | - |
| Dysgeusia | 2 (40·0) | - | - | - | 2 (13·3) | 1 (6·7) | - | - |
| Vomiting | 1 (20·0) | - | - | - | 2 (13·3) | 1 (6·7) | - | - |
| Weight Decreased | - | - | 1 (20·0) | - | 3 (20·0) | - | - | - |
| Blurred Vision | - | - | - | - | 1 (6·7) | 1 (6·7) | - | - |
| Constipation | - | - | - | - | - | 2 (13·3) | - | - |
| ECG QT Prolonged | - | - | 1 (20·0) | - | 1 (6·7) | - | - | - |
| Failure to Thrive | - | - | 1 (20·0) | - | - | 1 (6·7) | - | - |
| Hyponatremia | - | - | 1 (20·0) | - | - | - | 1 (6·7) | - |
| Sepsis | - | - | - | 1 (20·0) | - | - | - | 1 (6·7) |
| **Hematologic** | **G1** | **G2** | **G3** | **G4** | **G1** | **G2** | **G3** | **G4** |
| Neutropenia | - | - | - | 2 (40·0) | - | 1 (6·7) | - | 3 (20·0) |
| Anemia | - | - | 1 (20·0) | - | - | - | 3 (20·0) | 1 (6·7) |
| Thrombocytopenia | - | - | - | 1 (20·0) | - | - | 1 (6·7) | 2 (13·3) |
| Leukopenia | - | - | - | 1 (20·0) | 1 (6·7) | - | 1 (6·7) | 1 (6·7) |

**SUPPLEMENTAL FIGURES**

**
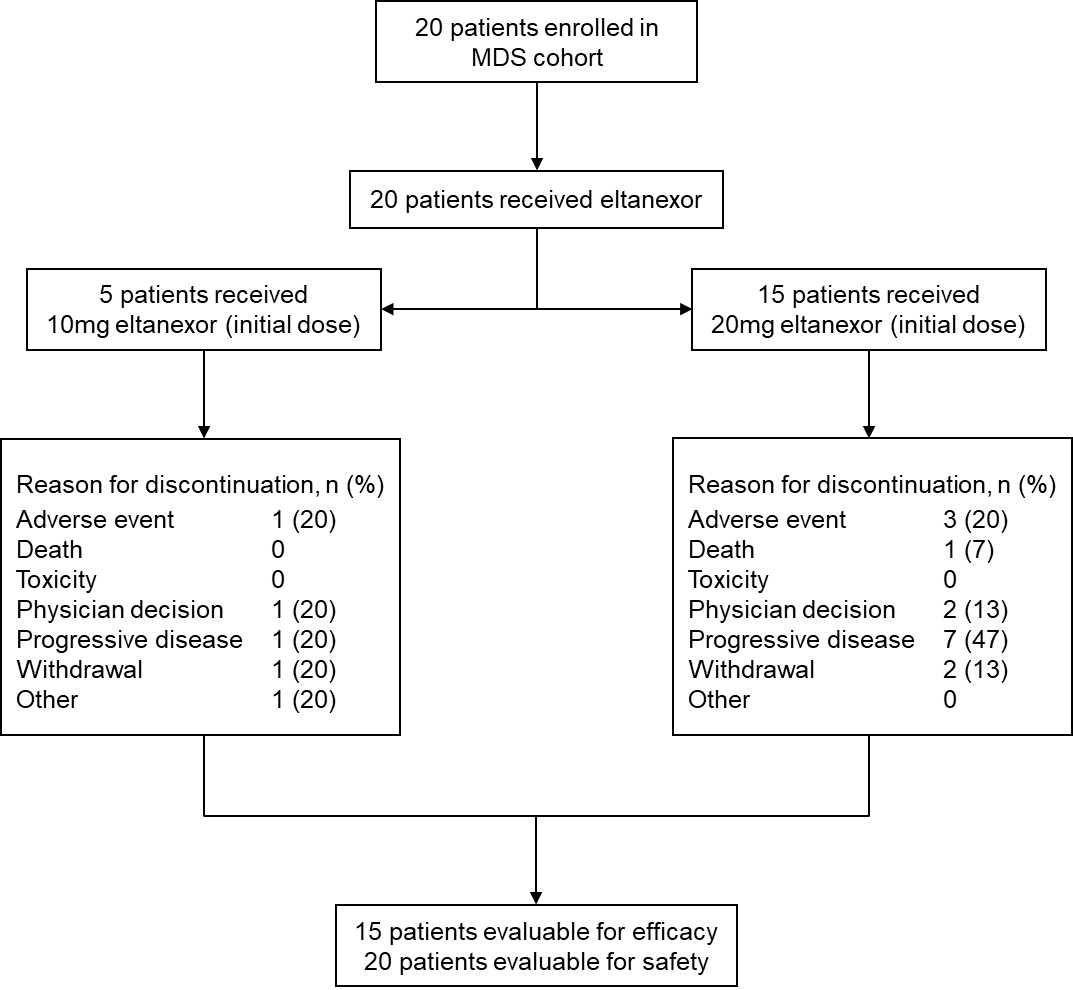
**

**Figure S1: Trial Profile.** Modified intention-to-treat analysis included all initial patients treated with 20mg eltanexor, regardless of dose reductions, and additional patients enrolled at 10mg dosage. Five patients on 20mg discontinued the study before the cycle three, day one assessment and were thus not evaluable for efficacy, but were included in safety analyses. MDS = myelodysplastic syndrome

**Blast % Change**

**800**

**600**

**400**

**200**

**0**

**0**

**5.0**

**2.5**

**7.5**

**10.0**

**Months from C1D1**


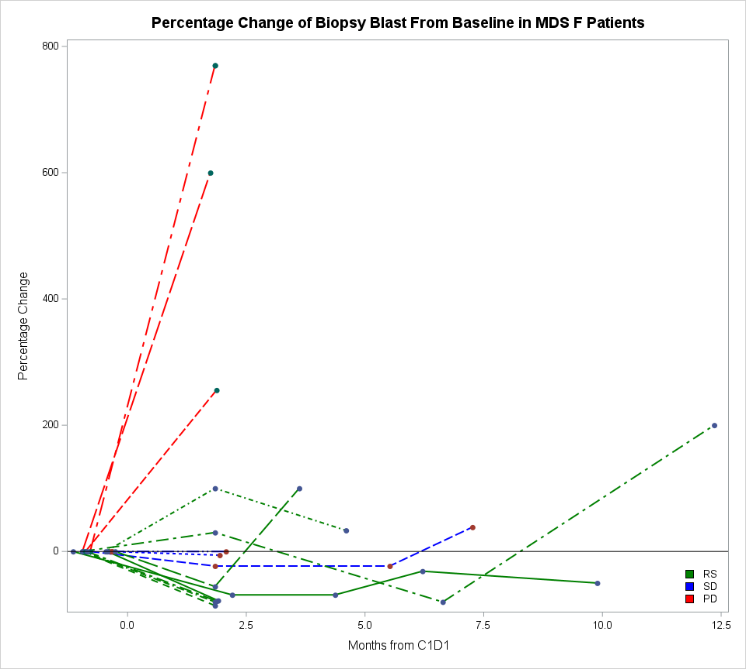


**12.5**

**A**

**Neutrophil % Change**

**400**

**300**

**200**

**100**

**0**

**0**

**4**

**2**

**6**

**8**

**Months from C1D1**


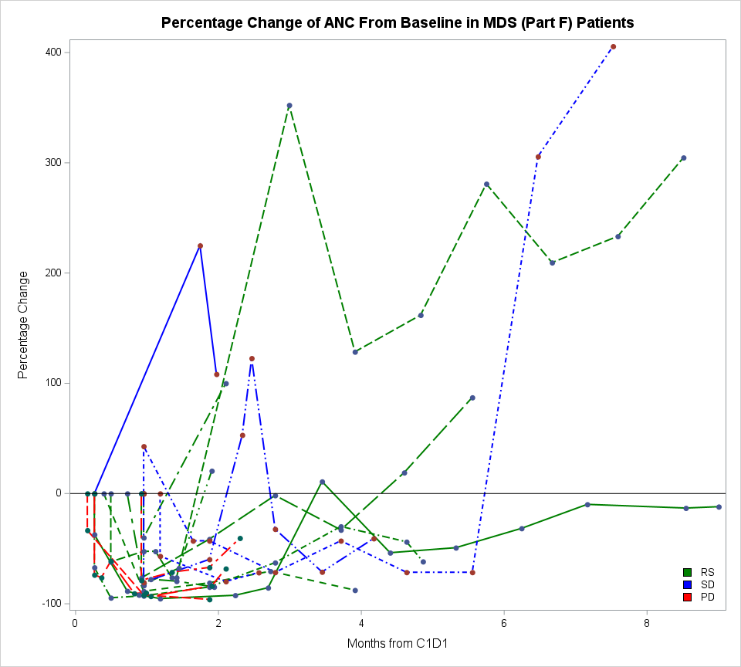


**-100**

**B**


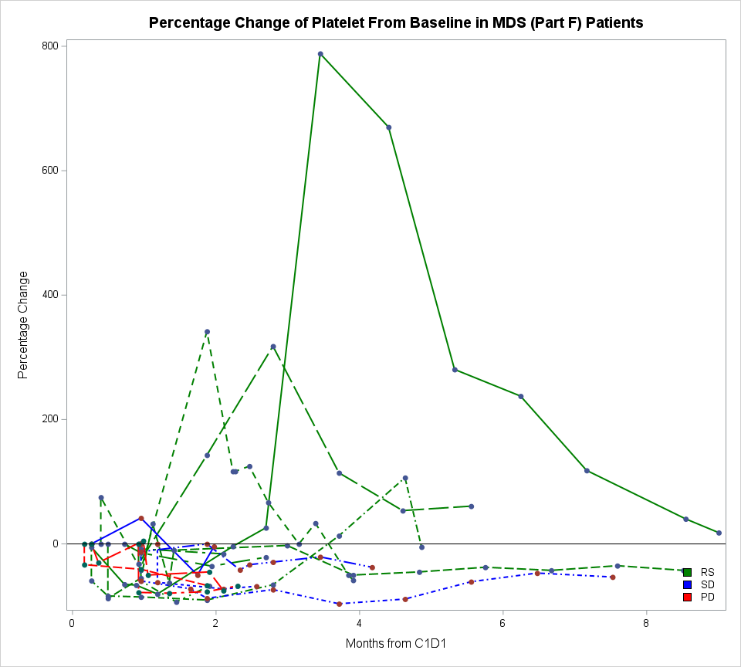


**Platelet % Change**

**800**

**600**

**400**

**200**

**0**

**0**

**4**

**2**

**6**

**8**

**Months from C1D1**

**C**

**Figure S2: Reduction in Leukemic Bone Marrow Blasts Corresponds with Increased Levels of Activated Neutrophils and Platelets*.*** Hematologic analysis of the efficacy evaluable population (n=15) among patients who achieved a response (RS) (*green*), defined as marrow complete remission + hematologic improvement, had SD (*blue*), and PD (*red*). Values presented for A) leukemic bone marrow blasts, B) activated neutrophil counts and C) platelet counts over time. MDS = myelodysplastic syndrome; RS = responders (marrow complete remission + hematologic improvement); SD = stable disease; PD = progressive disease; C1D1 = cycle 1 day 1
